# Immunoelectron microscopy findings in a patient with C3 glomerulonephritis 

**DOI:** 10.5414/CNCS111091

**Published:** 2023-06-08

**Authors:** Masato Sawamura, Naoki Sawa, Hiroki Mizuno, Yuki Oba, Daisuke Ikuma, Akinari Sekine, Masayuki Yamanouchi, Eiko Hasegawa, Tatsuya Suwabe, Masanori Suzuki, Kei Kono, Keiichi Kinowaki, Kenichi Ohashi, Takashi Ehara, Yoichiro Ikeda, Toshihiro Sawai, Yoshifumi Ubara

**Affiliations:** 1 Nephrology Center and Department of Rheumatology,; 2 Department of Pathology,; 3 Okinaka Memorial Institute for Medical Research, Toranomon Hospital,; 4 Department of Human Pathology, Tokyo Medical and Dental University, Tokyo,; 5 Department of Histopathology, Shinshu University School of Medicine, Matsumoto,; 6 Division of Nephrology and Endocrinology, the University of Tokyo Hospital, Tokyo, and; 7 Department of Pediatrics, Shiga University of Medical Science, Otsu, Japan

**Keywords:** C3 glomerulonephritis, immunoelectron microscopy, dense deposit disease, light microscopy, immunofluorescence microscopy

## Abstract

We performed a kidney biopsy in a 36-year-old man to evaluate microscopic hematuria and proteinuria. Light microscopy showed increased mesangial matrix and partial swelling of the glomerular basement membrane (GBM), and immunofluorescence showed positive staining only for C3. Immunoelectron microscopy showed that gold particle-labeled C3 was localized in the electron-dense and moderately electron-dense deposits shown by electron microscopy in the mesangium, the thickened GBM near the paramesangium, and the thickened distal portion of the GBM but was not localized in the non-thickened GBM. Gold-labeled immunoglobulin G, κ, and λ were not seen. C3 glomerulonephritis was more evident in gold-labeled electron microscopy, which further clarified the localization of C3 deposition.

## Introduction 

C3 glomerulopathy is classified on the basis of morphological differences in kidney biopsy specimens into two types of disease, C3 glomerulonephritis (C3GN) and dense deposit disease (DDD). C3GN and DDD can be difficult to distinguish from each other on light microscopy and immunofluorescence (IF) examination. However, electron microscopy shows mesangial and/or subendothelial, intramembranous, and subepithelial deposits in C3GN and dense osmiophilic deposits along the glomerular basement membrane (GBM) and in the mesangium in DDD. Both C3GN and DDD can be distinguished from immune complex-mediated glomerulonephritis by the lack of immunoglobulin (Ig) staining on IF [1]. However, it remains difficult to distinguish between the two diseases clinically and genetically [2]. 

Here, we attempted to prove that C3 is involved in the glomerular lesions in C3GN by examining the deposition sites of gold-labeled C3 by immunoelectron microscopy. 

## Case report 

A 36-year-old Japanese man was admitted to our hospital for evaluation of microscopic hematuria and proteinuria. The patient had a 10-year history of urinary abnormality, but he had not undergone close examination. He had no family history of kidney disease. 

On admission, the patient was 172 cm tall and weighed 77 kg; his blood pressure was 128/76 mmHg; temperature, 36.0 °C; and heart rate, 65 beats per min. A physical examination showed no abnormalities. The complete blood count was as follows (Table 1): erythrocytes, 5.03 × 10^6^/µL; hemoglobin, 14.7 g/dL; leucocytes, 6,000/µL; and thrombocytes, 28.4 × 10^4^/µL. The results of blood chemistry tests were as follows (Table 1): total protein, 6.1 g/dL; albumin, 3.8 g/dL; serum urea nitrogen, 20 mg/dL; serum creatinine, 1.26 mg/dL; estimated glomerular filtration rate (eGFR), 53.8 mL/min/1.73m^2^; IgG, 660 mg/dL (reference range, 870 – 1,700 mg/dL); IgA 155 mg/dL (reference range, 110 – 410 mg/dL); IgM, 74 mg/dL (reference range, 33 – 190 mg/dL); C3 85 mg/dL (reference range, 86 – 160 mg/dL), C4 29 mg/dL (reference range, 17 – 45 mg/dL); and CH50 60 U/mL (reference range, 30 – 40 U/mL). Tests for antinuclear antibody and cryoglobulins were negative, urinary protein excretion was 4.0 g/day, and the urinary sediment contained 30 – 49 red cells per high-power field. C3 nephritis factor measured using a previously published method was increased to 20.6% (normal value, < 12.0%), but anti-complement factor H (CFH) auto-autoantibody was negative. CFH, C3, and complement factor B (CFB) gene mutations were not evaluated [3], and kidney biopsy was performed. 

## Kidney biopsy 

**Figure 1.**
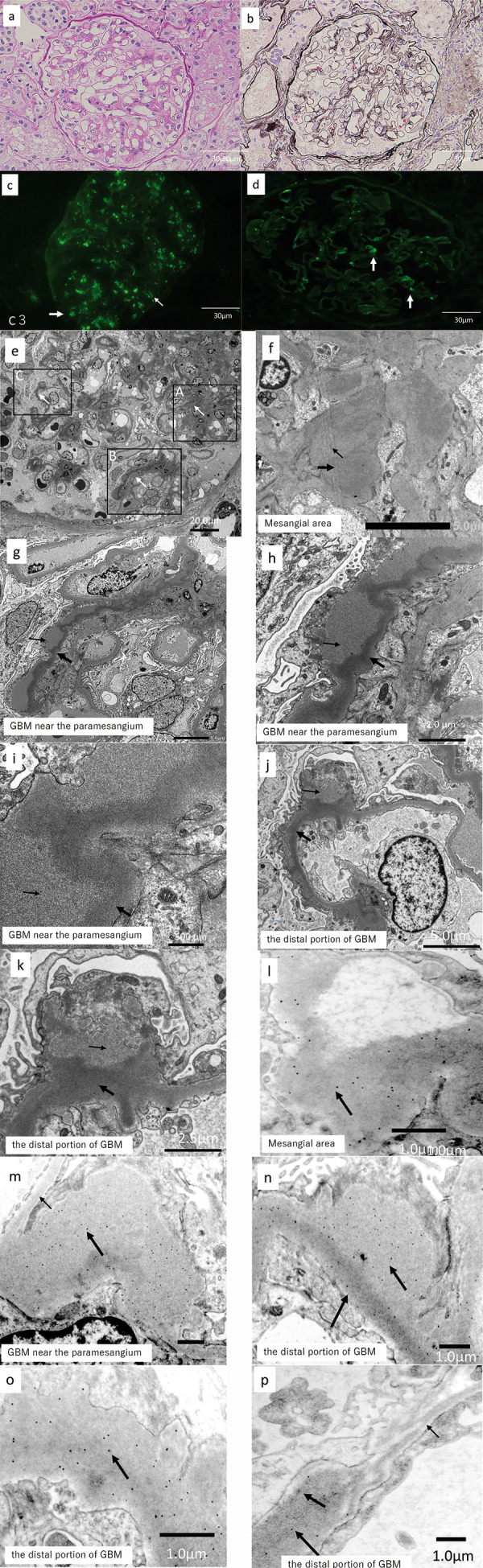
Microscopic analysis of the kidney biopsy specimen. a, b: Light microscopy showed partial thickening of the glomerular basement membrane (GBM) and a partial increase in the area of mesangial matrix. a: Periodic acid-Schiff staining; original magnification × 200; b: Periodic acid methenamine silver staining; original magnification × 200; c: Immunofluorescence microscopy showed mesangial dotted (large arrow) and peripheral linear (small arrow) staining for C3. Original magnification × 200; d: C4d staining showed positive findings in only a few glomeruli. Original magnification × 200. e – k: Electron microscopy. e: Weakly magnified images showing deposits (white arrow). Original magnification × 1,500; f: Strongly magnified images of square A of Figure 1d showing two types of deposits in the mesangial area: electron-dense deposits (large arrow) and moderately electron-dense deposits (small arrow). Original magnification × 9,000; g – i: Strongly magnified images of square B of Figure 1d showing deposits in the GBM near the paramesangium; deposits had a linear electron-dense structure (large arrow) on the endothelial side and were a moderately electron-dense structure (small arrow) on the epithelial side. g: Original magnification × 4,500; h: Original magnification × 9,000; i: original magnification × 18,000; j – k: Strongly magnified images of square C of Figure 1d showing deposits in the distal portion of the GBM consisting of linear and evenly distributed electron-dense deposits (large arrow) on the endothelial side and hump-shaped moderately electron-dense deposits (small arrow) on the epithelial side. j: Original magnification × 4,500; k: Original magnification × 9,000; l – p: Immunoelectron microscopy; l: Deposits in the mesangial area: Gold particle-labeled C3 was seen (black dots). Original magnification × 10,000; m: Deposits in the GBM near the paramesangium: Gold particle-labeled C3 was noted throughout the thickened GBM but was not present in normal GBM (small arrow). Original magnification × 5,000; n: Deposits in thickened GBM: Gold particle-labeled C3 was noted throughout the thickened GBM (arrow). Original magnification × 5,000; o: Irregularly thickened GBM: Gold particle-labeled C3 was noted throughout the thickened GBM (arrow). Original magnification × 10,000; p. Thickened and non-thickened GBM; Gold particle-labeled C3 was noted throughout the thickened GBM (large arrow) but was not present in normal-range GBM (small arrow). Original magnification × 5,000.

Light microscopy examination of a biopsy specimen containing 20 glomeruli revealed global sclerosis of 2 glomeruli. Partial thickening of the GBM and a partial increase in the area of the mesangial matrix were also noted (Figure 1a, b). IF microscopy showed mesangial dotted and peripheral linear staining for C3 (Figure 1c), but staining was negative for IgG, IgA, IgM, C4, and C1q. C4d staining showed positive findings in only a few parts of glomeruli (Figure 1d), although staining was not as strong as for C3. 

On electron microscopy, electron-dense deposits were seen mainly in the mesangial area, the GBM near the paramesangium, and the distal portion of the GBM (Figure 1e). In the mesangial area, both electron-dense and moderately electron-dense deposits were seen (Figure 1f). Deposits in the GBM near the paramesangium consisted of linear electron-dense structures on the endothelial side and moderately electron-dense deposits on the epithelial side (Figures 1g, h, i). Deposits in the distal portion of the GBM consisted of linear electron-dense deposits on the endothelial side and hump-shaped, moderately electron-dense deposits on the epithelial side (Figures 1j, k). 

Immunoelectron microscopy performed according to a previously published method [4] showed that gold particle-labeled C3 was localized in the electron-dense and moderately electron-dense deposits seen on electron microscopy; however, it was not present in non-thickened GBM (Figures 1l, m, n, o, p). On the other hand, no gold-labeled IgG, κ, or λ were found in the above areas. These findings indicate that the deposits identified by electron microscopy reflected C3 deposition. 

## Clinical course 

**Figure 2.**
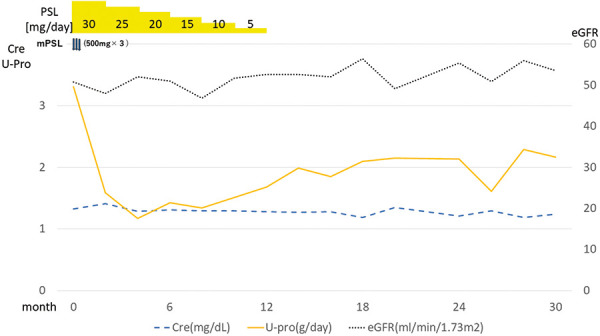
Clinical course.

We initiated treatment with methylprednisolone at a dose of 500 mg/day for 3 days, followed by oral prednisolone at a dose of 30 mg/day for 1 year. Proteinuria decreased temporarily after the treatment, but after 2 years, it remained at ~ 2 g/day. Renal function remained unchanged (Figure 2). 

## Discussion 

We presented a case of C3GN evaluated by immunoelectron microscopy. Sethi et al. [1] evaluated the amount of complement and immunoglobulins in patients with C3GN by using laser dissection to cut glomeruli from specimens taken by kidney biopsy and performing mass spectrometry to determine the glomerular proteomic profile. Extensive deposition of C3 and C9 was seen, with a mean of 51.3 and 13.6 spectra, respectively, and C5, C6, C7, and C8 were also present, with a mean of 8.5, 3.5, 4.8, and 9.3 spectra, respectively; however, little C1, C2, and C4 and few immunoglobulins and κ and λ light chain proteins were seen. These results may indicate that the classical complement pathway or heavy and light chains of immunoglobulins are less involved in C3GN [1]. 

In the present case, we used immunoelectron microscopy to examine the deposition sites of gold-labeled C3. Other researchers have used immunoelectron microscopy techniques to evaluate the involvement of immunoglobulins and complement in glomerular lesions and the involvement of the membrane attack complex of complement in IgA nephropathy, light chain crystalline tubulopathy, and other diseases [4, 5, 6, 7, 8, 9]. These studies are briefly described below. 

Ehara and Shigematsu [4] used immunoelectron microscopy to examine whether mast cells in the interstitium in IgA nephritis show tryptase or chymase activity; Herrera et al. [5] examined the localization of the light chain component in monoclonal light chain-related renal diseases; Gu et al. [6] examined seven cases of light chain crystal deposition (κ light chain-related); Hinglais et al. [7] examined the localization of the C5b-9 complex of complement in various glomerular diseases; Miyamoto et al. [8] examined the localization of the membrane attack complex of complement in patients with primary IgA nephropathy; and Nakamura et al. [9] examined the presence of monoclonal Bence-Jones protein and free κ light chains in a patient with light chain proximal tubulopathy. 

C3GN is one of the C3 glomerulopathies associated with monoclonal gammopathy. Monoclonal gammopathy of renal significance refers to all kidney disorders caused by a monoclonal immunoglobulin secreted by a nonmalignant B-cell clone. Although immunoelectron microscopy may be important for determining the involvement of immunoglobulins and complement in C3GN, to our knowledge no previous reports have described immunoelectron microscopy findings in this disease [10]. 

Recently, Sethi et al. [11] reported that C4d deposition shown by in the glomerulus may be a diagnostic marker for differentiating between immunocomplex-related glomerulonephritis and C3GN/DDD. Subsequently, Singh et al. [12] conducted a similar study using paraffin specimen and reported that patients with C3GN/DDD showed mild to trace-level C4d deposition; in some cases, the group obtained a clear, positive image. We performed C4d staining by IF method in the present case and found scattered positive findings in only a few parts of glomeruli, although staining was not as strong as that of C3. This result may indicate that part of the classical pathway (with C1q deposition) or lectin pathway (without C1q deposition) of complement activation is partially involved in C3GN. 

In most cases of immunocomplex-related glomerulonephritis, tissue damage is mainly caused by the association of immunoglobulins such as IgA, IgG, and IgM with complement proteins such as C3. Electron-dense deposits identified by electron microscopy have been thought to be consistent with the positive images seen by IF staining, but it remains unclear whether this is indeed the case. 

On the other hand, C3GN is unique in that it involves solely C3 deposition without any immunoglobulins. In C3GN, the electron-dense region seen on electron microscopy is considered to be the lesion responsible for the disease. However, the result of gold immunoelectron staining in our case suggests that C3 deposits are not limited to electron-dense areas in the GBM and mesangium but are also found in thickened moderately electron-dense areas of the GBM and mesangium, even in areas without electron-dense deposits. Staining for C3 deposits was negative in the non-thickened areas. It can be inferred that the responsible lesion in C3GN identified by gold immunoelectron staining is different from the findings in conventional electron microscopy. 

In conclusion, we performed immunoelectron microscopy on a kidney biopsy specimen from a 36-year-old man diagnosed with C3GN. Gold particle-labeled C3 was localized in thickened areas on the electron-dense and moderately electron-dense deposits shown by electron microscopy in the mesangium, the GBM near the paramesangium, and the distal portion of the GBM; however, it was not seen in non-thickened areas. Staining for gold-labeled IgG, κ, and λ was negative. This case shows that in C3GN, gold particle-labeled electron microscopy may help elucidate the pathogenesis of C3GN. 

## Statement of ethics 

The present report was produced in conformity with the Declaration of Helsinki, and the patient gave consent for this case report to be published. 

## Funding 

The authors did not receive support from any organization for the submitted work. 

## Conflict of interest 

The authors declare that they have no conflicts of interest. 

**Table 1.** Laboratory data.

	At kidney biopsy	Nomal range
White blood cell (/µL)	2,300	3,200 – 7,900
Red blood cell (10^6^/µL)	3.68	3.7 – 5.0
Hemoglobin (g/dL)	11.4	11.3 – 15.0
Platelet (×10^3^/µL)	14.3	155 – 350
Total protein (g/dL)	5.6	6.9 – 8.4
Albumin (g/dL)	3.8	4.1 – 5.1
Urea nitrogen (mg/dL)	59	8 – 21
Creatinine (mg/dL)	4.4	0.6-1.0
eGFR (mL/min/1.73m^2^)	17	≥ 90
C-reactive protein (CRP)	0.13	< 0.3
Aspartate aminotransferase (IU/L)	20	11 – 38
Alanine transaminase (IU/L)	31	6 – 50
Lactate dehydrogenase (IU/L)	526	103 – 190
Alkaline phosphatase (U/L)	247	38 – 113
Myoglobin (µg/L)	2,560	< 106
Prothrombin time (%)	89	> 75
Activated partial thromboplastin time (sec)	24.4	27 – 40
D dimer	7.06	< 0.99
ADAMTS13 activity(%)	39	78 – 157
ADAMTS13 activity inhibitor (BU/mL)	0.5	< 0.5
Complement activities 50 (U/mL)	58	30 – 46
Complement 3 (mg/dL)	62	86 – 160
Complement 4 (mg/dL)	20	17 – 45
Renin activity (ng/mL/h)	21	0.2 – 2.9
Aldosterone concentration (ng/dL)	26	2.9 – 15.9
IgG (mg/dL)	649	870 – 1,700
IgA (mg/dL)	31	110 – 410
IgM (mg/dL)	22	46 – 260
Antinuclear antibody (ANA)	320	< 40
Anti-double-stranded DNA antibody (IU/mL)	7	< 12
Anti-SS-A antibody (U/mL)	< 1	< 1
Anti-SS-B antibody (U/mL)	< 1	< 1
Anti-cardiolipin antibody	Negative	Negative
Myeloperoxidase anti-neutrophil cytoplasmic antibodies (MPO-ANCA)	Negative	Negative
Anti-proteinase-3 anti-neutrophil cytoplasmic antibodies (PR3-ANCA)	Negative	Negative
Hepatitis B virus (HBV) antibody	Negative	Negative
Anti-hepatitis C virus (HCV) antibody	Negative	Negative
Interleukin-6 (pg/mL)	684	< 4.0
Vascular endothelial growth factor (pg/mL)	6,406	140 – 659
Urinary RBC sediment(/HPF)	< 1	< 1
Urinary protein (g/day)	2.38	< 0.15
Urinary Bence-Jones protein	Negative	Negative
